# Properties of Two Enterovirus Antibodies that are Utilized in Diabetes Research

**DOI:** 10.1038/srep24757

**Published:** 2016-04-19

**Authors:** Giuseppe Maccari, Angelo Genoni, Silvia Sansonno, Antonio Toniolo

**Affiliations:** 1 Center for Nanotechnology and Innovation, Italian Institute of Technology, Pisa, Italy; 2 Laboratory of Medical Microbiology, Department of Biotechnology and Life Sciences, University of Insubria, Varese, Italy; 3 Department of Medical Sciences, University of Foggia, Foggia, Italy

## Abstract

Human enteroviruses (EVs) comprise >100 different types. Research suggests a non-chance association between EV infections and type 1 diabetes. Immunohistochemical studies with the anti-EV antibody 5D-8.1 have shown that the EV capsid antigen is present in pancreatic islet cells of diabetic subjects. When it was noticed that 5D-8.1 may cross-react with human proteins, doubt was casted on the significance of the above histopathologic findings. To address this issue, properties of EV antibodies 5D-8.1 and 9D5 have been investigated using peptide microarrays, peptide substitution scanning, immunofluorescence of EV-infected cells, EV neutralization assays, bioinformatics analysis. Evidence indicates that the two antibodies bind to distinct non-neutralizing linear epitopes in VP1 and are specific for a vast spectrum of EV types (not for other human viruses). However, their epitopes may align with a few human proteins at low *expected values*. When tested by immunofluorescence, high concentrations of 5D-8.1 yelded faint cytoplasmic staining in uninfected cells. At reduced concentrations, both antibodies produced dotted staining only in the cytoplasm of infected cells and recognized both acute and persistent EV infection. Thus, the two monoclonals represent distinct and independent probes for hunting EVs in tissues of patients with diabetes or other endocrine conditions.

Several recent reports point to enterovirus (EV) infections as key environmental triggers of type 1 diabetes (T1D)^1^,^2^,^3^,^4^,^5^,^6^,^7^. Conclusions are based on multiple proofs that include the histopathologic detection of EV antigens/genome in the islets of Langerhans of diabetics at different stages of the disease^8^,^9^,^10^,^11^,^12^. Recent findings suggest that EVs are causing chronic low-level infection in the islet cells of newly diagnosed T1D patients^12^.

EVs are small, non-enveloped, single-strand positive-sense RNA viruses belonging to the family *Picornaviridae*. The EV capsid is composed of four structural proteins named VP1, VP2, VP3, and VP4. The main structural differences among VP1, VP2, and VP3 lie in the loops that connect the beta-strands with the N- and C-terminal sequences extending from the beta-barrel domain^13^. These amino acid (AA) sequences give each EV its distinct morphology and antigenicity. The VP4 component lies on the inner surface of the shell and is essential for virion stability. Evolution has resulted in a large number of antigenically distinguishable members that have been categorized as EV “serotypes”^13^. Not considering human rhinoviruses, the EV genus contains human agents of the A, B, C, D, and unnamed species that, together, comprise 109 different types.

Each of the serotypes correlates with a specific immune response of the host and protection from disease. The serotype-specific protective immune response is directed to the capsid proteins VP1, VP2, and VP3, as the VP4 has no role in the interaction with neutralizing antibodies (i.e., those directed to variable regions of surface capsid proteins). Non-neutralizing antibodies recognizing VP regions that are conserved among different EV types are also produced. The significance of the non-neutralizing antibody response is currently under investigation^14^.

Immunization with different EV types allowed to produce a variety of monoclonal antibodies (MAbs) that are either type-specific (i.e., responsible of virus neutralization) or directed to conserved regions of capsid proteins^15^,^16^,^17^,^18^. Reactivity of the latter antibodies may be limited to sets of different EV types or may be directed to a wider range of EV types. Though molecular methods are held to be more informative than classical serologic methods for virus identification^19^, “pan-EV antibodies” capable of reacting with all or with the majority of EV types remain desirable reagents for detecting these agents both in the diagnosis of infectious diseases^20^,^21^,^22^,^23^ and in immunohistochemistry^24^.

The greater part of immunohistochemical studies in which a conserved region of enteroviral VP1 has been detected in the islets of Langerhans of T1D cases employed the pan-EV MAb 5D-8.1^10^,^25^. This MAb has been produced in mice immunized with inactivated coxsackievirus B5 (CV-B5) and has been shown, by immunostaining, to react with multiple EV types^18^,^20^,^23^. The 5D-8.1 epitope in VP1 has been partially characterized using competition assays with synthetic peptides and demonstrated to be EV-specific^26^. However, subsequent studies suggested that MAb 5D-8.1 may also recognize human proteins, including an isoform of creatine kinase and a mitochondrial ATP synthase^27^. Comprehensibly, these results casted doubts on the conclusions of previous immunohistochemical studies of pancreatic tissue in T1D cases^25^,^28^.

Due to the relevance of MAb 5D-8.1 in diabetes research, we re-investigated this antibody in parallel with the pan-EV MAb 9D5 that is used for diagnosing EV infections in virology laboratories^20^,^21^. MAb 9D5 has been obtained from mice immunized with inactivated CV-B3 and shown to react with multiple EV types^20^.

Reactivity of the two MAbs was defined with the help of innovative microarray technology, substitution scan of peptide epitopes, immunostaining of acutely and persistently infected cell lines, neutralization assays. The MAbs’ epitopes were then examined versus the human proteome and versus proteins of diverse viral agents in order to delineate their specificity and define possible cross-reactivities.

## Results

### Epitopes of Mab 5D-8.1 and MAb 9D5

**Figure 1:**
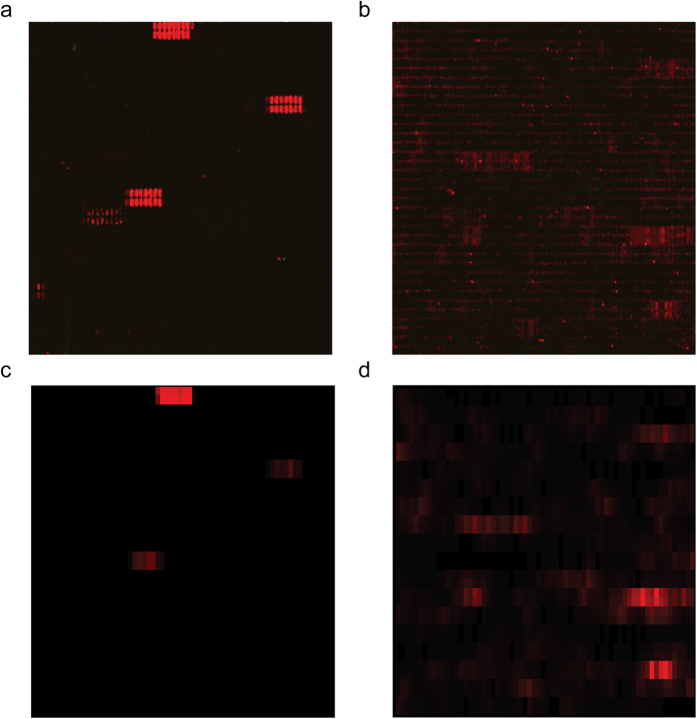
Spot patterns produced by MAbs 5D-8.1 (**a**) and 9D5 (**b**) with overlapping peptides of the VP1 protein of the following viruses: CV-B1, CV-B4, E-30, CV-A1. Microarray signals were converted to a matrix representation: 5D8.1 (**c**), and 9D5 (**d**). Background noise was reduced by multiplying the signal with the moving average of the intensity plot.

Secondary goat anti-mouse IgG Ab did not show background interactions with antigen-derived peptides. As shown in Fig. 1a,c, MAb 5D-8.1 gave defined spots for the VP1 sequence of CV-B1, CV-B4, E-30, and reduced reactivity with CV-A1. The peptide scan indicated IPALTAVETGHT as the consensus sequence containing the epitope of MAb 5D-8.1. Comparable signals were produced by MAb 9D5 (Fig. 1b,d) and attributed to the consensus motif SIGNAYSMFYDG. Thus, relative to the VP1 sequence of CV-B4 (GI: 61031)^29^, the sequence containing the epitope of 5D-8.1 was close to the N-terminus at AA residues 28–39, whereas that of 5D9 was located towards the C-terminus at residues 187–198. Substitution scan of peptide IPALTAVETGHT against MAb 5D-8.1 allowed to delimit the antibody binding site to a conserved core motif 4-IPALTAAET-12. AA positions 4I, 7L and 8T showed the highest degree of sequence conservation with a nearly complete loss in binding of MAb 5D-8.1 upon exchange by other amino acids. AA positions 5P and 11E were well-conserved, exchange by Q and by H, respectively, caused a 60–80% loss in antibody binding. Compared to this, AA positions 6A and 10A exhibited a slightly higher susceptibility for substitution by selected amino acids with a maximal decrease of 50% of spot intensities. 9A and 12T showed the highest tolerance for exchange by other amino acids. Replacement by F (9A) and A (12T) was accepted without loss in antibody binding.

### Three dimensional analysis of the VP1 protein and the two MAb epitopes

**Figure 2:**
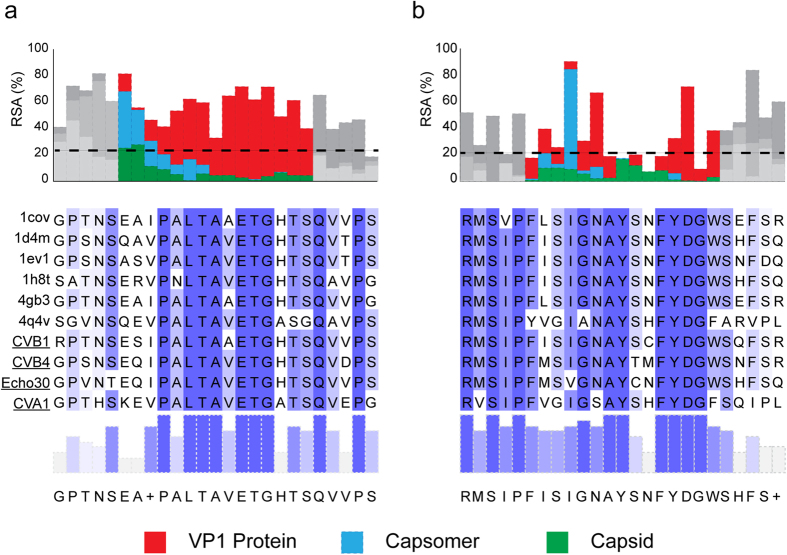
Solvent-Accessible Surface Area (SASA) of the VP1 capsid protein calculated at different organization levels and expressed as Relative Solvent Accessibility (RSA) a quantity that varies between 0 and 100 (Red: VP1 protein; blue: capsomer; green: capsid). Only the epitope region is colored. The alignment of the two epitope sequences is highlighted in shades of blue to represent the conservation level. Panel (**a**): MAb 5D-8.1; Panel (**b**): MAb 9D5.

**Figure 3:**
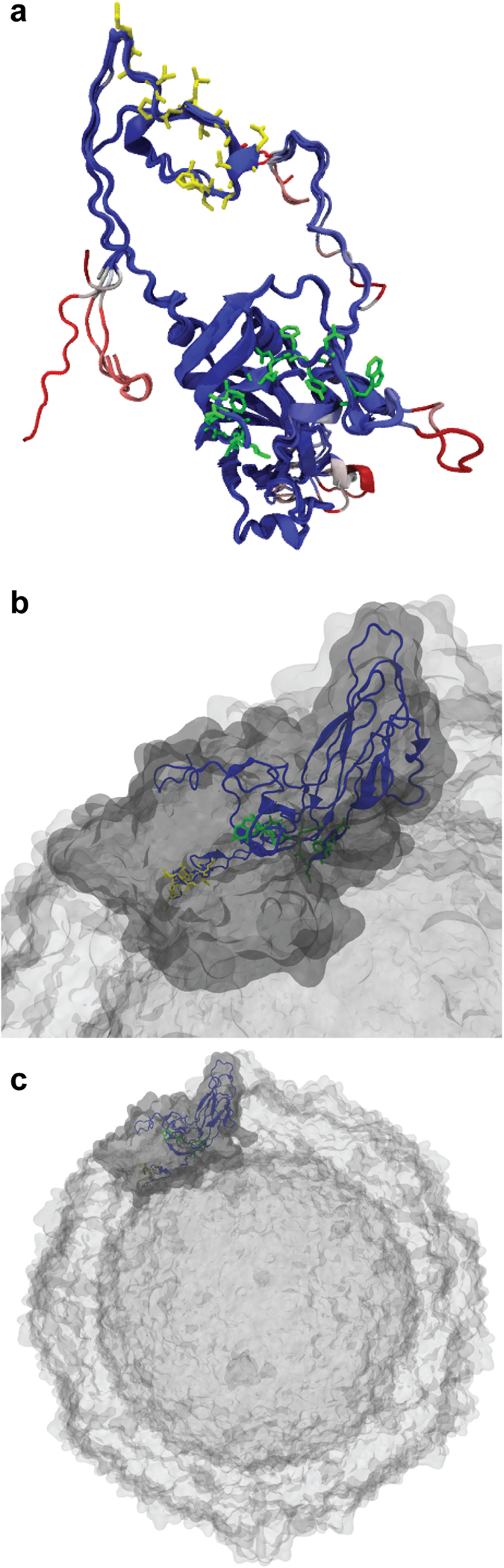
Structural alignment of the capsid protein VP1. The resolved VP1 structure of 6 enterovirus types (CV-B1, CV-B3, CV-A24, CV-A21, E-1, E-7) was aligned in order to identify highly conserved regions. (**a**) Soluble VP1: the 5D-8.1 epitope (yellow) and the 9D5 epitope (green) are indicated. (**b**) VP1 assembled into an enterovirus capsomere. (**c**) Localization of VP1 within the enterovirus capsid.

For each viral capsid organization level, the Solvent-Accessible Surface Area (SASA) was calculated in order to estimate the degree-of-burial of antibody epitopes within the protein. The resolved structures of six reference enterovirus strains were obtained from the RCSB database and a 1.4 Å sphere probe was used to represent a water molecule. The exposed surface area was first calculated, and then normalized with the maximum allowed solvent-accessible area^30^. Normalized SASA values take the form of Relative Solvent Accessibility (RSA), a quantity which varies between 0 (for completely buried residues) and 100 (for maximally exposed residues). Results are summarized in Fig. 2. The alignment of the VP1 regions recognized by the two MAbs is shown in Fig. 3a. The target residues are mainly accessible in the monomeric form for the two epitopes (N-terminal 5D-8.1, yellow; C- terminal 9D5, green). The exposed residues are highly conserved among different EV types, evidencing their importance in the capsid assembly process. Localization of the VP1 protein within the capsomere (Fig. 3b) shows that the 5D-8.1 epitope is located in a domain where exposed residues are stabilized by a beta sheet structure. Figure 3c shows the two epitopes in the VP1 protein assembled into the capsid.

### Detection of MAb epitope sequences among human and viral proteins

The predicted reactivity of antibodies 5D-8.1 and 9D5 with human proteins and viral agents was explored using BLASTp queries vs. the human proteome and viruses. Results are summarized in Table 1. It is deduced that the two MAbs may recognize linear targets producing significant alignments with sufficiently low E values. Interestingly, MAb 5D-8.1 produced significant alignments for creatine kinase U-type and a mitochondrial ATP synthase, among other proteins. These targets may represent the antigens indicated as cross-reactive by Korsgren and collaborators in 2013^27^. Similarly, MAb 9D5 may be expected to bind to a variety of human proteins, including the leucine-rich repeat-containing protein 66. However, much better alignments (i.e., much lower E values) have been obtained for enteroviruses, rhinoviruses, and agents of the Rabovirus and Sapelovirus genera that represent the most recent members of the *Picornaviridae* family^31^,^32^.

The expected reactivity of MAbs 5D-8.1 and 9D5 with different EV types of the A, B, C, D species is shown in Table 2. Whereas reactivity of the two MAbs is largely equivalent, 5D-8.1 has a wider coverage of the A species as compared to MAb 9D5. Scattered cases of complimentary specificity also occur, indicating that the combined use of both antibodies could widen the detection range in diagnostic/immunohistochemical procedures.

### Immunostaining of enterovirus-infected cell cultures

**Figure 4:**
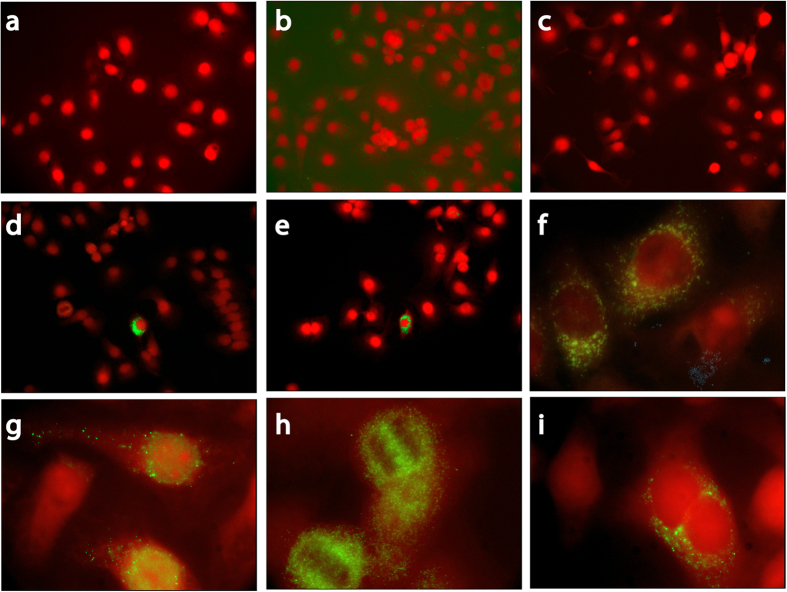
Indirect immunofluorescence of the human AV3 cell line: staining of enterovirus-infected cells with MAbs 9D5 and 5D-8.1 (green; counterstaining, Evans blue). Uninfected cells (top panel), cells acutely infected with CV-B4 (middle panel), cells persistently infected with CV-B1pc (lower panel).At the concentration of 5 μg/ml, MAb 9D5 did not produce fluorescence in uninfected cells (**a**). 5D-8.1 produced fine granular cytoplasmic fluorescence in uninfected cells at the concentration of 1 μg/ml (**b**). Background staining disappeared when this antibody was used at concentrations ≤1 μg/ml (**c**). AV3 cells 4 hrs post-infection with CV-B4: staining by 5D-8.1 (**d**,**e**), or 9D5 (**f**).AV3 cells undergoing persistent infection by the CV-B1pc strain. 5D-8.1: dotted cytoplasmic fluorescence (**g**). 9D5: VP1 staining frequently seen in cells showing mitotic bars (**h**) or dividing (**i**). Original magnification: 20× (**a**–**e**) or 100× (**f**–**i**).

In uninfected cells (AV3 and LLC-MK_2_ lines) MAb 9D5 did not produce fluorescence even at the concentration of 5 μg/ml (Fig. 4a), while 5D-8.1 yielded fine perinuclear and cytoplasmic fluorescence when used at the concentration of 1 μg/ml (Fig. 4b), but not at concentrations ≤1 μg/ml (Fig. 4c). The two investigated MAbs produced dotted cytoplasmic fluorescence in human and monkey cells acutely infected with CV-B4 (Fig. 4d–f). Fine dotted fluorescence was also seen in AV3 cells undergoing persistent infection by the CV-B1pc strain isolated from a case of pancreatic carcinoma (Fig. 4g–i). In persistent infection, VP1 was expressed frequently in cells showing mitotic bars or dividing (Fig. 4h,i). The slow infectious process was not accompanied by manifest CPE.

IIF was also used for investigating the inhibitory effects of peptides containing the epitopes of MAbs 5D-8.1 and 9D5 in the acute model of CV-B4 infection. Fluorescent staining by 5D-8.1 was totally inhibited by pre-incubation with the peptide SESIPALTAAETGHT (8 μg/ml), but not with peptide SIGNAYSNFYDG. The reverse was true for MAb 9D5: pre-incubation with SIGNAYSNFYDG (8 μg/ml), but not with SESIPALTAAETGHT, inhibited cytoplasmic fluorescence (data not shown). Thus, IIF confirmed that the two AA sequences encompass the relevant epitopes.

### Enterovirus neutralization assays with MAbs 5D-8.1 and 9D5

The neutralizing activity of 5D-8.1 and 9D5 was explored against CV-B1 and CV-B4. As controls, horse antiserum against CV-B1 and the CV-B4-neutralizing MAb 356 were used. As shown in Table 3, the two pan-EV antibodies did not neutralize CV-B1 and CV-B4 (titer <1:8). As expected, control antibodies had high homotypic, but not heterotypic, neutralizing titers (anti CV-B1: 1:4096 vs. CV-B1; <1:8 vs. CV-B4 - anti CV-B4: 1:512 vs. CV-B4; <1:8 vs. CV-B1). Thus, the investigated monoclonals are devoid of neutralizing activity.

## Discussion

Validation of antibodies used to identify specific biomolecules is a critical issue in medicine^33^. To this end, a variety of methods can be used, but it is recommended that rather than relying on a single antibody, researchers should have the possibility of using pairs of antibodies designed to bind different parts of the same target protein. The case of MAb 5D-8.1 is remarkable in the context of diabetes research. In fact, numerous immunohistochemical studies with 5D-8.1 documented the presence of EV VP1 within the islets of Langerhans in a large proportion of T1D cases, but not in the pancreas of non-diabetic subjects^28^. These studies suggested that viral infection played a pathogenic role in T1D. In 2013, it was shown that MAb 5D-8.1 could bind human islet proteins, specifically the mitochondrial proteins creatine kinase B-type and ATP synthase beta subunit^27^. The finding triggered a reassessment of EV infection of pancreatic islets in T1D cases^34^. Two subsequent publications settled the issue in part, convening that - under appropriate staining conditions - MAb 5D-8.1 was an adequate probe for EV infection^25^,^28^.

As virologists, we set out to validate the binding of 5D-8.1 and 9D5 to the EV VP1 capsid component and to identify the possible cross reactivity of these antibodies both with human proteins and viral agents. Binding results and bioinformatics analyses confirmed that the epitopes of 5D-8.1 and 9D5 are distinct and located at the N- and C-terminal domains of VP1. Both antibodies are directed to conserved domains of a capsid protein of picornaviruses, and recognize the majority of EV types. However, they are not neutralizing, as expected for antibodies targeting conserved regions of the viral shell. Our data delineate the spectrum of EV types that each antibody binds to, thus confirming partial published results on the specificity of the two MAbs^18^,^20^,^24^,^26^. Bioinformatics analysis indicated that the two antibodies cover EV types of the A species less well than those of the B, C, D species (Table 2). Since the binding spectra are not identical, the combined use of MAbs 5D-8.1 and 9D5 should allow to cover almost all EV types. Notably, both antibodies are also predicted to cover Rabovirus and Sapeloviruses, animal viruses of the most recent genera within the *Picornaviridae* family. Immunofluorescence results confirmed that MAb 5D-8.1 (but not 9D5) may produce fine granular fluorescence in the cytoplasm of uninfected human and monkey cells. This, however, occurred only at elevated antibody concentrations (i.e., >1 μg/ml). The observation is in line with the lack of absolute specificity of EVs reported by Korsgren and collaborators^27^. We could however confirm that, when adequately diluted, the antibody produces specific staining of different EV types in cultured cells^25^ without fluorescent signals in uninfected cells. Of interest to diabetes research, the linear epitopes of both MAbs bear only marginal similarity with the human proteome, with a few possible exceptions. In particular, the 5D-8.1 epitope bears similarity with creatine kinase U-type (*E value*, 3.0), ATP synthase mitochondrial F1 complex assembly factor (*E value*, 12), creatine kinase B-type (*E value*, 66).

Notably, 9D5 - that has been used for a long time in diagnostic virology^18^,^20^ –does not produce background staining in uninfected cultured cells and delineates clearly the expression of VP1 in acutely- and persistently-infected cells. Immunofluorescent staining of cultured cells cannot be compared directly with that of immunocytochemistry. In formalin-fixed, paraffin-embedded samples, antigen retrieval is mandatory for VP1 detection^25^ (suggesting that protein denaturation may favor antibody binding). In contrast, staining of cultured cells with either of these MAbs gives excellent results upon acetone or paraformaldehyde fixation (i.e., procedures not causing protein unfolding). Whether the two MAbs can bind to VP1 in its native configuration^35^ within live virus particles is currently under investigation.

Finally, in persistently-infected cells, expression of VP1 was frequently seen in proliferating cells with both 5D-8.1 and 9D5. The finding may not be accidental. In fact, the cellular factor 68-kDa Src-associated protein in mitosis (Sam68) has been recently shown to interact with the EV IRES during infection, thus enhancing translation of virus proteins^36^. This aspect merits further attention due to its possible impact on EV pathogenesis^36^

In conclusion, both MAbs bind to the VP1 capsid protein of EVs and of phylogenetically-related picornaviruses. Epitopes are located in distinct stretches of the VP1 protein. The MAbs recognize both acute and persistent infection in cultured cells, and are devoid of EV-neutralizing activity. Thus, these distinct and independent probes will be useful for confirming histopathologic and virology data that indicate EV infection of islet cells as having a pathogenic role in diabetes. Further virus searches in diabetes and other endocrine diseases are expected.

## Methods

### Identification of linear epitopes in VP1 sequences of four enterovirus types

The linear epitopes of MAbs 5D-8.1 and 9D5 in the VP1 capsid protein of enteroviruses were mapped using peptide microarray technology^37^. The N- and C-termini of VP1 sequences of four enterovirus types [coxsackievirus B1 (CV-B1; K4N918), coxsackievirus-B4 (CV-B4; S5PU54), echovirus-30 (E-30; Q9YLK0), and coxsackievirus-A1 (CV-A1; Q9YLP4)] were elongated by neutral GSGSGSG linkers to avoid truncated peptides. Elongated sequences were linked to a single artificial sequence. The elongated artificial sequence was translated into 15 AA peptides with a peptide-peptide overlap of 14 amino acids and bound to duplicate spots of silica microarrays. The resulting microarrays covered peptides of all four sequences (1,181 different peptides in duplicate). For control, microarrays were framed by HA (YPYDVPDYAG) and FLAG (DYKDDDDKGG) peptides (104 spots). Rockland blocking buffer MB-070 (VWR International Frankfurt, DE), PBS plus 0.05% Tween 20, PBS-Tween plus 10% Rockland blocking buffer were used for blocking, washing, and incubation procedures, respectively. Mouse mAb 5D-8.1 (IgG2a; Dako, Cernusco sul Naviglio, Italy) and 9D5 (IgG3; Millipore, Livingstone, UK) were incubated at 10 μg/ml in the microarray for 16 h at 4 °C with shaking. Goat anti-mouse IgG-DyLight680 (New England Biolabs, Frankfurt, DE) served as secondary antibody and was incubated for 30 min at room temperature. HA and FLAG control peptides framing the microarray were subsequently stained with MAb anti-HA-DyLight680 (red) and MAb anti-FLAG-DyLight800 (green). Light emission was read with a LI-COR Odyssey Infrared Imaging System (resolution 21 μm; 700 nm; LI-COR Biosciences, Bad Homburg, DE). Staining with MAbs anti-HA and anti-FLAG confirmed the assay quality and microarray integrity (scanning intensities red/green 7/7).

### Substitution scan of the peptide SESIPALTAAETGHT against MAb 5D-81

Peptide array synthesis and binding detection were performed by PEPperPRINT GmbH (PEPperPRINT, Heidelberg, Germany) as reported^38^,^39^. Permutation scans were carried out on the peptide SESIPALTAAETGHT (containing the MAb 5D-8.1 epitope) and on its variants. In the permutation scan, the effect on binding of replacing each of the 15 peptide positions by standard L-amino-acids was analyzed. Each microarray contained 286 peptides printed in duplicate and was framed by HA and FLAG control peptides. One peptide microarray copy was pre-stained with the secondary goat anti-mouse IgG (H+L) DyLight680 antibody (red) in the presence of the monoclonal anti-HA (12CA5)-DyLight800 antibody (green) followed by read-out at scanning intensities of 7/7 (red/green). Incubation of a second peptide microarray with MAb 5D-8.1 (1 μg/ml) was followed by staining with the secondary antibody in the presence of the monoclonal anti-HA (12CA5)-DyLight800 control antibody and then read-out at scanning intensities of 7/7 (red/green). Finally, the FLAG control peptides framing the peptide arrays were stained as additional internal quality control to confirm the assay quality and the peptide microarray integrity (scanning intensities red/green: 7/7).

Quantification of spot intensities and peptide annotation were based on 16-bit gray scale tiff files at scanning intensities of 7/7. Microarray image analysis was done with PepSlide Analyzer. A software algorithm broke down fluorescence intensities of each channel and spot into raw, foreground, background signal and calculated the standard deviation of median foreground intensities. Based on averaged median foreground intensities, an intensity map was generated and interactions in the peptide map were highlighted by an intensity color code with red for high and white for low spot intensities. To provide an in-depth view on the substitution scan, a heat map of the peptide microarray was generated as well as a substitution matrix and an AA plot reflecting the AA preferences at a given position. Data sets were correlated with peptide and intensity maps to analyze the substitution pattern in consideration of conserved/variable amino acids and possible AA exchanges.

The substitution matrix highlighted the preference for a given AA by color codes (red: preferred AA; light blue: less preferred AAs) and was calculated by dividing the spot intensity of a given peptide (e.g., 1-YPYDVQDYA-9) by the averaged spot intensities of all 20 peptides that were substituted at the same position (1-YPYDVQDYA-9). The AA plot was calculated by dividing the spot intensity of a given peptide (1-YPYDVQDYA-9) by the spot intensity of the wild type epitope (1-YPYDVQDYA-9). The position of an AA at a given position thus reflected the intensity ratio compared to the AA of the native epitope at the same position.

### Cell lines, viruses, indirect immunofluorescence (IIF) and neutralization assays

The human cell lines AV3, HeLa, RD and the monkey line LLC-MK_2_ (ECAAC, Salisbury, UK) were cultured in DMEM/F12 medium supplemented with 10% FCS plus penicillin/streptomycin. EV types CV-B1 (Conn-5), CV-B4 (J.V.B.), E-30 (Bastianni), CV-A1 (Tompkins) obtained from ATCC (LGC Standards, Sesto San Giovanni, Italy) were amplified *in vitro*, titrated and stored at −70 °C. For acute infection, subconfluent cultures were infected at a multiplicity of infection of 0.5 and incubated 3–6 hours. Persistent infection was investigated in the AV3 cell line that had been chronically infected with CV-B1pc (a virus strain isolated from tissue collateral to pancreatic carcinoma). AV3 cells carrying CV-B1pc do not show evident CPE, maintain apparently normal replication rate, and release minimal amounts of virus in the medium as seen by RT-PCR and virus titration (≤100 TCID_50_/ml; manuscript in preparation). Persistently infected cells were at the 10^th^–20^th^ passage.

For IIF, cells were cultured in 4-well Millicell EZ Slides (Merck, Vimodrone, Italy). Cell monolayers were fixed in 4% paraformaldehyde in PBS (r.t., 30 min), washed 3× in PBS-1% FCS, permeabilized with Triton X100 (0.05% in PBS; 10 min), washed 3× in PBS-1% FCS, briefly immersed in distilled water and dried out. Fixed slides were incubated overnight at 4 °C with 0.5, 1, or 5 μg/ml of mouse pan-EV MAb 5D-8.1 (IgG2a) or MAb 9D5 (IgG3). Staining was achieved with the secondary antibodies FITC-labeled goat anti-mouse IgG H+L (Merck), Alexa Fluor 488 goat anti-mouse IgG2a, or Alexa Fluor 594 goat anti-mouse IgG3 (ThermoFisher, Monza, Italy). ProLong antifade (ThermoFisher) was used as mounting medium. Pictures were taken with either a Nikon E800 Eclipse microscope (Nikon, Firenze, Italy) or a Leica TCS SP8 confocal microscope (Leica, Milano, Italy).

Indirect immunofluorescence (IIF) inhibition assays of MAbs 5D-8.1 and 9D5 have been performed using different concentrations of the synthetic peptides SESIPALTAAETGHT (PEPperPRINT) and SIGNAYSCFYDG (Sigma-Aldrich, Milano, Italy) that encompass the epitopes of 5D-8.1 and 9D5, respectively. Each MAb (1 μg/ml) was pre-incubated with increasing concentrations (0, 2, 8, 32 μg/ml) of either the SESIPALTAAETGHT or the SIGNAYSCFYDG peptide (2 hrs at r.t.). Then, IIF staining was performed as above.

Neutralization assays were performed as reported^40^. The following MAbs/antisera were used: MAb 5D-8.1, MAb 9D5, anti-CV-B4 MAb 356^41^, horse anti-CV-B1 serum (ATCC). Briefly, antibody dilutions in complete DMEM/F12 medium were made in triplicate in 96-well flat bottom plates. Then, 100 tissue culture infectious doses_50_ (TCID_50_) of CV-B1 or CV-B4 were dispensed into each well and mixed with antibody dilutions or control medium. After 1 hr incubation at r.t., 10^4^ LLC-MK_2_ cells were added to each well. Cytopathic effect (CPE) was read at 6–7 days with an inverted microscope. Antibody titer is defined as the highest antibody dilution capable of preventing CPE.

### Bioinformatics analysis

A list of complete EV capsid structures were obtained from the *advanced search* method of the RSCB server^42^, and a selection of the resulting data were downloaded as monomer PDB files (1COV, 1H8T, 1D4M, 1EV1, 4GB3, 4Q4V). For each monomer structure, the complete capsid was assembled based on its BIOMT REMARK included in the PDB file. The Visual Molecular Dynamics software (VMD)^43^ has been used for visualization, computation and analysis of structural data. The Solvent-Accessible Surface area (SASA) was calculated for the VP1 monomer, the capsomer, the capsid. For each epitope, BLASTp queries were performed in public databases (*Homo sapiens*, Viruses, *Picornavirales,* ssRNA viruses, enteroviruses, rhinoviruses). A BLAST program employing the SEG algorithm^44^ was used to filter low complexity regions from proteins before executing a database search. The BLASTp results that produced significant alignments of the two MAb epitopes with human proteins or viral agents are reported along with the *Expected*(*E*) *value. E value* is used as a measure of epitope specificity. The lower the *E-value*, or the closer it is to zero, the more “significant” the match is. Analysis of the predicted reactivity of the two MAbs with the different EV types is based on the alignments of epitope sequences with the VP1 sequence of EVs. A cut off value of 70% AA identity has been used.

## Additional Information

**How to cite this article**: Maccari, G. *et al.* Properties of Two Enterovirus Antibodies that are Utilized in Diabetes Research. *Sci. Rep.* **6**, 24757; doi: 10.1038/srep24757 (2016).

## Figures and Tables

**Table 1:** Significant alignments of human proteins and viral agents with the epitopes of monoclonal antibodies 5D-8.1 and 9D5^1^.

Target proteins	MAb 5D-8.1		MAb 9D5	
	Sequence PALTAVETGHT containing theMab epitope	BLASTp query*E value* (range)	Sequence SIGNAYSNFYDG containing theMab epitope	BLASTp query*E value* (range)
*Homo sapiens*	Creatine kinase U-type; protein sel-1 homolog 1 isoform 2 precursor; ATP synthase mitochondrial F1 complex assembly factor; cAMP-specific 3′,5′-cyclic phosphodiesterase 4D; olfactory receptor 52I1; DDB1- and CUL4-associated factor 4-like protein 1; interferon regulatory factor 2-binding protein 2 isoform B; receptor-interacting serine/threonine-protein kinase 4; ATP synthase subunit gamma, mitochondrial; serine/threonine-protein kinase mTOR; Creatine kinase B-type	3.0–66	Leucine-rich repeat-containing protein 66; sodium/potassium/calcium exchanger 6, mitochondrial precursor; fin bud initiation factor homolog precursor; cullin-7 isoforms; inter-alpha-trypsin inhibitor heavy chain H3 preproprotein; FK506-binding protein 15; regenerating islet-derived protein 3-alpha precursor; Synaptotagmin 12; leucine-rich repeat-containing protein 36; heterogeneous nuclear ribonucleoprotein U-like protein 2; IQ and AAA domain-containing protein 1-like	1.1–35
Animal viruses	Enterovirus species A, B, C, D, E, F, G, H, J; Rhinovirus A, B, C; Rabovirus; Sapelovirus	8 × 10^−5^–1.7	Enterovirus species A, B, C, D, E, F, G, H, J; Rhinovirus A, B, C; Rabovirus; Sapelovirus; Saffold virus; Coronavirus	9 × 10^−6^–1.5
Other viruses	Bacterial phages	36–70	Bacterial phages	11–41

^1^BLASTp queries performed in public databases. Taxid: *Homo sapiens*, Viruses, Picornavirales, ssRNA viruses, Enteroviruses, Rhinoviruses.

**Table 2:**
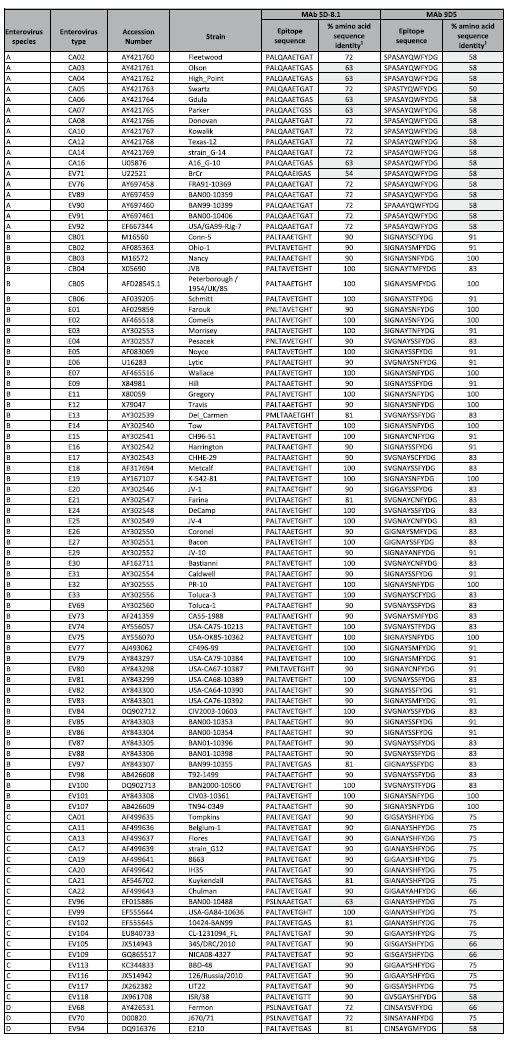
Epitopes of MAbs 5D-8.1 and 9D5: significant alignments with the VP1 protein of enterovirus types belonging to the A, B, C, D species.

^1^Enterovirus types showing less than 70% sequence identity with the epitope sequence are highlighted in gray.

**Table 3:** Enterovirus neutralization assays. Results for monoclonal antibodies 5D-8.1 and 9D5.

Antibody/antiserum	Neutralizing titer^1^	
	CV-B1	CV-B4
Pan-enterovirus MAb 5D-8.1	<8	<8
Pan-enterovirus MAb 59D5	<8	<8
Anti-CV-B4 MAb 356 (control)	<8	512
Anti-CV-B1 horse antiserum (control)	4096	<8

^1^Neutralizing titer expressed as the inverse of the greatest dilution giving a positive result.

## References

[b1] Yeung W.-C. G., Rawlinson W. D. & Craig M. E. Enterovirus infection and type 1 diabetes mellitus: systematic review and meta-analysis of observational molecular studies. BMJ 342, d35, doi: 10.1136/bmj.d35 (2011). 21292721 PMC3033438

[b2] Oikarinen M. *et al.* Type 1 diabetes is associated with enterovirus infection in gut mucosa. Diabetes 61, 687–91 (2012). 22315304 10.2337/db11-1157 PMC3282798

[b3] Oikarinen S. *et al.* Virus antibody survey in different European populations indicates risk association between coxsackievirus B1 and type 1 diabetes. Diabetes 63, 655–62 (2014). 24009257 10.2337/db13-0620

[b4] Salvatoni A. *et al.* Intrafamilial spread of enterovirus infections at the clinical onset of type 1 diabetes. Pediatr. Diabetes 14, 407–16 (2013). 23763622 10.1111/pedi.12056

[b5] Alidjinou E. K. *et al.* Monocytes of Patients with Type 1 Diabetes Harbour Enterovirus RNA. Eur. J. Clin. Invest. 45, 918–24 (2015). 26108863 10.1111/eci.12485

[b6] Taylor K., Hyöty H., Toniolo A. & Zuckerman A. J. Diabetes and Viruses. (Springer: New York, , 2013), doi: 10.1007/978-1-4614-4051-2.

[b7] Nagafuchi S. & Toniolo A. Viral diabetes: virus diabetogenicity and host susceptibility. Immunoendocrinology 2, e1026, doi: 10.14800/ie.1026 (2015).

[b8] In’t Veld P. Insulitis in the human endocrine pancreas: does a viral infection lead to inflammation and beta cell replication? Diabetologia 54, 2220–2 (2011). 21701817 10.1007/s00125-011-2224-3

[b9] Kobayashi T., Nishida Y., Tanaka S. & Aida K. Pathological changes in the pancreas of fulminant type 1 diabetes and slowly progressive insulin-dependent diabetes mellitus (SPIDDM): innate immunity in fulminant type 1 diabetes and SPIDDM. Diabetes. Metab. Res. Rev. 27, 965–70 (2011). 22069294 10.1002/dmrr.1237

[b10] Richardson S. J., Leete P., Bone A. J., Foulis A. K. & Morgan N. G. Expression of the enteroviral capsid protein VP1 in the islet cells of patients with type 1 diabetes is associated with induction of protein kinase R and downregulation of Mcl-1. Diabetologia 56, 185–93 (2013). 23064357 10.1007/s00125-012-2745-4

[b11] Bissel S. J. *et al.* Coxsackievirus B4 myocarditis and meningoencephalitis in newborn twins. Neuropathology 34, 429–37 (2014). 24702280 10.1111/neup.12121 PMC4188796

[b12] Krogvold L. *et al.* Detection of a low-grade enteroviral infection in the islets of langerhans of living patients newly diagnosed with type 1 diabetes. Diabetes 64, 1682–7 (2015). 25422108 10.2337/db14-1370

[b13] Fields B. N., Knipe D. M. & Howley P. M. Fields virology. (Wolters Kluwer Health/Lippincott Williams & Wilkins, 2013). ISBN/ISSN: 9781451105636.

[b14] Alidjinou E. K., Sané F., Engelmann I. & Hober D. Serum-dependent enhancement of coxsackievirus B4-induced production of IFNα, IL-6 and TNFα by peripheral blood mononuclear cells. J. Mol. Biol. 425, 5020–31 (2013). 24120940 10.1016/j.jmb.2013.10.008

[b15] Chen Z. *et al.* Cross-neutralizing human anti-poliovirus antibodies bind the recognition site for cellular receptor. Proc. Natl. Acad. Sci. USA 110, 20242–7 (2013). 24277851 10.1073/pnas.1320041110 PMC3864303

[b16] Ku Z. *et al.* Single Neutralizing Monoclonal Antibodies Targeting the VP1 GH Loop of Enterovirus 71 Inhibit both Virus Attachment and Internalization during Viral Entry. J. Virol. 89, 12084–95 (2015). 26401034 10.1128/JVI.02189-15 PMC4645313

[b17] Kiener T. K., Jia Q., Meng T., Chow V. T. K. & Kwang J. A novel universal neutralizing monoclonal antibody against enterovirus 71 that targets the highly conserved ‘knob’ region of VP3 protein. PLoS Negl. Trop. Dis. 8, e2895, doi: 10.1371/journal.pntd.0002895 (2014). 24875055 PMC4038473

[b18] Miao L. Y. *et al.* Monoclonal antibodies to VP1 recognize a broad range of enteroviruses. J. Clin. Microbiol. 47, 3108–13 (2009). 19710278 10.1128/JCM.00479-09 PMC2756915

[b19] Muir P. *et al.* Molecular typing of enteroviruses: current status and future requirements. The European Union Concerted Action on Virus Meningitis and Encephalitis. Clin. Microbiol. Rev. 11, 202–27 (1998). 9457433 10.1128/cmr.11.1.202 PMC121380

[b20] Yagi S., Schnurr D. & Lin J. Spectrum of monoclonal antibodies to coxsackievirus B-3 includes type- and group-specific antibodies. J. Clin. Microbiol. 30, 2498–501 (1992). 1328290 10.1128/jcm.30.9.2498-2501.1992 PMC265534

[b21] Leonardi G. P. & Costello P. Use of monoclonal antibody blends in the identification of enteroviral aseptic meningitis. Curr. Microbiol. 28, 49–52 (1994).

[b22] Yousef G. E., Mann G. F., Brown I. N. & Mowbray J. F. Clinical and research application of an enterovirus group-reactive monoclonal antibody. Intervirology 28, 199–205 (1987). 2835329 10.1159/000150017

[b23] Yousef G. E., Brown I. N. & Mowbray J. F. Derivation and biochemical characterization of an enterovirus group-specific monoclonal antibody. Intervirology 28, 163–70 (1987). 2836335 10.1159/000150012

[b24] Zhang H. *et al.* Localization of enteroviral antigen in myocardium and other tissues from patients with heart muscle disease by an improved immunohistochemical technique. J. Histochem. Cytochem. 48, 579–84 (2000). 10769041 10.1177/002215540004800501

[b25] Richardson S. J. *et al.* Evaluation of the fidelity of immunolabelling obtained with clone 5D8/1, a monoclonal antibody directed against the enteroviral capsid protein, VP1, in human pancreas. Diabetologia 57, 392–401 (2014). 24190581 10.1007/s00125-013-3094-7

[b26] Samuelson A., Forsgren M. & Sällberg M. Characterization of the recognition site and diagnostic potential of an enterovirus group-reactive monoclonal antibody. Clin. Diagn. Lab. Immunol. 2, 385–6 (1995). 7545081 10.1128/cdli.2.3.385-386.1995 PMC170165

[b27] Hansson S. F., Korsgren S., Pontén F. & Korsgren O. Enteroviruses and the pathogenesis of type 1 diabetes revisited: cross-reactivity of enterovirus capsid protein (VP1) antibodies with human mitochondrial proteins. J. Pathol. 229, 719–28 (2013). 23335350 10.1002/path.4166

[b28] Richardson S. J. *et al.* Detection of enterovirus in the islet cells of patients with type 1 diabetes: what do we learn from immunohistochemistry? Reply to Hansson SF, Korsgren S, Pontén F et al [letter]. Diabetologia 57, 647–9 (2014). 24429580 10.1007/s00125-014-3167-2

[b29] Jenkins O., Booth J. D., Minor P. D. & Almond J. W. The complete nucleotide sequence of coxsackievirus B4 and its comparison to other members of the Picornaviridae. J. Gen. Virol. 68, 1835–48 (1987). 3037008 10.1099/0022-1317-68-7-1835

[b30] Tien M. Z., Meyer A. G., Sydykova D. K., Spielman S. J. & Wilke C. O. Maximum allowed solvent accessibilites of residues in proteins. PLoS One 8, e80635, doi: 10.1371/journal.pone.0080635 (2013). 24278298 PMC3836772

[b31] Oberste M. S., Maher K. & Pallansch M. A. Genomic evidence that simian virus 2 and six other simian picornaviruses represent a new genus in Picornaviridae. Virology 314, 283–93 (2003). 14517081 10.1016/s0042-6822(03)00420-3

[b32] Ng T. F. *et al.* Rabovirus: a proposed new picornavirus genus that is phylogenetically basal to enteroviruses and sapeloviruses. Arch. Virol. 160, 2569–75 (2015). 26168710 10.1007/s00705-015-2523-y

[b33] Baker M. Antibody anarchy: A call to order. Nature 527, 545–551 (2015). 26607547 10.1038/527545a

[b34] Coppieters K. T. & von Herrath M. Antibody cross-reactivity and the viral aetiology of type 1 diabetes. J. Pathol. 230, 1–3 (2013). 23389883 10.1002/path.4174

[b35] Roivainen M., Piirainen L., Rysä T., Närvänen A. & Hovi T. An immunodominant N-terminal region of VP1 protein of poliovirion that is buried in crystal structure can be exposed in solution. Virology. 195, 762–765 (1993). 8393243 10.1006/viro.1993.1427

[b36] Zhang H., Song L., Cong H. & Tien P. Nuclear protein Sam68 interacts with the Enterovirus 71 Internal Ribosome Entry Site and positively regulates viral protein translation. J. Virol. 89, 10031–43 (2015). 26202240 10.1128/JVI.01677-15 PMC4577883

[b37] Nesterov A. *et al.* Peptide arrays with a chip. Methods Mol. Biol. 669, 109–24 (2010). 20857361 10.1007/978-1-60761-845-4_9

[b38] Nominé Y., Choulier L., Travé G., Vernet T. & Altschuh D. Antibody Binding Selectivity: Alternative Sets of Antigen Residues Entail High-Affinity Recognition. PLoS One 10, e0143374, doi: 10.1371/journal.pone.0143374 (2015). 26629896 PMC4667898

[b39] Vernet T. *et al.* Spot peptide arrays and SPR measurements: throughput and quantification in antibody selectivity studies. J. Mol. Recognit. 28, 635–44 (2015). 25960426 10.1002/jmr.2477

[b40] Toniolo A., Onodera T., Jordan G., Yoon J. W. & Notkins A. L. Virus-induced diabetes mellitus. Glucose abnormalities produced in mice by the six members of the Coxsackie B virus group. Diabetes 31, 496–9 (1982). 6759266 10.2337/diab.31.6.496

[b41] Prabhakar B. S., Haspel M. V., McClintock P. R. & Notkins A. L. High frequency of antigenic variants among naturally occurring human Coxsackie B4 virus isolates identified by monoclonal antibodies. Nature 300, 374–376 (1982). 6183593 10.1038/300374a0

[b42] Rose P. W. *et al.* The RCSB Protein Data Bank: views of structural biology for basic and applied research and education. Nucleic Acids Res. 43 (Database issue), D345–56 (2015). 25428375 10.1093/nar/gku1214 PMC4383988

[b43] Humphrey W., Dalke A. & Schulten K. VMD: visual molecular dynamics. J. Mol. Graph. 14, 33–8, 27–8 (1996). 8744570 10.1016/0263-7855(96)00018-5

[b44] Wootton J. C. & Federhen S. Statistics of local complexity in amino acid sequences and sequence databases. Comput. Chem. 17, 149–163 (1993).

